# Clinical, ultrasound and molecular biomarkers for early prediction of large for gestational age infants in nulliparous women: An international prospective cohort study

**DOI:** 10.1371/journal.pone.0178484

**Published:** 2017-06-01

**Authors:** Matias C. Vieira, Lesley M. E. McCowan, Alexandra Gillett, Lucilla Poston, Elaine Fyfe, Gustaaf A. Dekker, Philip N. Baker, James J. Walker, Louise C. Kenny, Dharmintra Pasupathy, on behalf of the SCOPE Consortium

**Affiliations:** 1 Division of Women’s Health, Women’s Health Academic Centre, King’s College London and King’s Health Partners, London, United Kingdom; 2 Núcleo de Formação Específica em Ginecologia e Obstetrícia, Escola de Medicina, Pontifícia Universidade Católica do Rio Grande do Sul, Porto Alegre, Brazil; 3 Department of Obstetrics and Gynaecology, Faculty of Medical and Health Sciences, University of Auckland, Auckland, New Zealand; 4 NIHR Biomedical Research Centre at Guy’s and St Thomas’ NH Foundation Trust and King’s College London, King’s College London, London, United Kingdom; 5 Women's and Children's Division Lyell McEwin Hospital, University of Adelaide, Adelaide, South Australia, Australia; 6 College of Medicine, Biological Sciences & Psychology, University of Leicester, Leicester, United Kingdom; 7 Department of Obstetrics and Gynaecology, Leeds Institute of Biomedical & Clinical Sciences, University of Leeds, Leeds, United Kingdom; 8 The Irish Centre for Fetal and Neonatal Translational Research (INFANT), Department of Obstetrics and Gynaecology, University College Cork, Cork University Maternity Hospital, Wilton, Cork, Ireland; University of Bristol, UNITED KINGDOM

## Abstract

**Objective:**

To develop a prediction model for term infants born large for gestational age (LGA) by customised birthweight centiles.

**Methods:**

International prospective cohort of nulliparous women with singleton pregnancy recruited to the Screening for Pregnancy Endpoints (SCOPE) study. LGA was defined as birthweight above the 90^th^ customised centile, including adjustment for parity, ethnicity, maternal height and weight, fetal gender and gestational age. Clinical risk factors, ultrasound parameters and biomarkers at 14–16 or 19–21 weeks were combined into a prediction model for LGA infants at term using stepwise logistic regression in a training dataset. Prediction performance was assessed in a validation dataset using area under the Receiver Operating Characteristics curve (AUC) and detection rate at fixed false positive rates.

**Results:**

The prevalence of LGA at term was 8.8% (n = 491/5628). Clinical and ultrasound factors selected in the prediction model for LGA infants were maternal birthweight, gestational weight gain between 14–16 and 19–21 weeks, and fetal abdominal circumference, head circumference and uterine artery Doppler resistance index at 19–21 weeks (AUC 0.67; 95%CI 0.63–0.71). Sensitivity of this model was 24% and 49% for a fixed false positive rate of 10% and 25%, respectively. The addition of biomarkers resulted in selection of random glucose, LDL-cholesterol, vascular endothelial growth factor receptor-1 (VEGFR1) and neutrophil gelatinase-associated lipocalin (NGAL), but with minimal improvement in model performance (AUC 0.69; 95%CI 0.65–0.73). Sensitivity of the full model was 26% and 50% for a fixed false positive rate of 10% and 25%, respectively.

**Conclusion:**

Prediction of LGA infants at term has limited diagnostic performance before 22 weeks but may have a role in contingency screening in later pregnancy.

## Introduction

Large for gestational age (LGA) is usually defined as birth weight above the 90^th^ centile and is associated with adverse perinatal outcomes [1]. Several reports, including observational studies and a meta-analysis of two small randomised controlled trials, assessed induction of labour for suspected large fetuses, and concluded that induction did not significantly reduce adverse outcomes [2, 3]. However, a recent large randomised controlled trial (RCT) of induction of labour versus expectant management in suspected LGA pregnancies demonstrated that induction of labour at 37–39 weeks was associated with a 68% reduction in related adverse outcomes [4]. In light of this evidence, new strategies are needed to improve antenatal identification of LGA infants.

At present in most settings, screening for LGA is based on abdominal palpation and/or fundal height measurement and in some cases referral for ultrasound, although this is not consistent practice. The estimated sensitivity of these clinical methods is between 9.7% and 16.6% [5–7]. Routine third trimester ultrasound in unselected populations has better performance in detecting abnormal growth however is not universal practice [8]. Development of reliable early pregnancy prediction models for LGA infants would offer the opportunity to undertake trials of interventions that may prevent fetal overgrowth (primary prevention) or could inform which women are more likely to benefit from a third trimester ultrasound and help direct resources. The latter would allow appropriate management of labour and delivery in order to reduce the likelihood of complications (secondary prevention).

Using data from the Screening for Pregnancy Endpoints (SCOPE) study, a prospective international cohort of nulliparous pregnant women, our group previously reported that LGA as defined by customised centiles, which adjusts for maternal ethnicity, height, early pregnancy weight, parity, gestation at delivery and infant sex, was more strongly associated with adverse perinatal outcomes compared to LGA defined by population centiles or birthweight above 4000g [9]. The aim of the present study was to assess the performance of early pregnancy factors for prediction of LGA at term defined by customised centiles.

## Methods

SCOPE is an international prospective cohort study involving centres in Auckland, New Zealand; Adelaide, Australia; London, Manchester and Leeds, UK; and Cork, Ireland. Ethical approval was obtained from local ethics committees (New Zealand AKX/02/00/364, Australia REC 1712/5/2008, London, Leeds and Manchester 06/MRE01/98 and Cork ECM5 (10) 05/02/08) and all women provided written informed consent prior to entering the study.

SCOPE recruited healthy nulliparous women with singleton pregnancies at 14–16 weeks between November 2004 and February 2011 [10]. Women were excluded if they were at high risk of preeclampsia, small for gestational age (SGA) or preterm birth because of underlying medical conditions, had at least three previous miscarriages or terminations of pregnancy, with major fetal anomaly or abnormal karyotype prior to recruitment, or those who received interventions that may modify pregnancy outcome. Extensive information was collected on socio-demographic and clinical characteristics, and blood samples were also obtained. The data collected and sample storage and analysis are described in detail elsewhere [11]. At 19–21 weeks, women returned for clinical assessment and a fetal ultrasound scan for biometry and uterine and umbilical artery Doppler waveform analysis. Women were followed up within 72 hours of delivery and data on pregnancy and neonatal outcome were collected [10].

The date of last menstrual period (LMP) was used to determine the estimated due date (EDD) which was then confirmed by ultrasound. The EDD was only corrected if (i) a scan performed before 16 weeks identified a difference of seven days or more or (ii) the 20 weeks scan identified a difference of 10 days or more between the scan EDD and the LMP EDD. If the EDD based on LMP was uncertain then the EDD was based on the scan. For the majority of participants (96%), an ultrasound before 16 weeks was available to confirm, correct, or assign the EDD.

### Outcomes of interest

A LGA infant born at term, was defined as an infant born at or beyond 37 weeks with a birthweight above the 90^th^ customised centile. Fetal growth above the 95^th^ customised centile was also explored. Customised centiles were calculated correcting for gestational age, maternal ethnicity, height and weight in early pregnancy, parity and infant sex [12].

### Exposures

The selection of clinical factors for prediction of LGA at term was based on a-priori hypothesis of biological plausibility and/or known association with LGA. Those included were maternal birthweight, maternal preterm birth, family history of diabetes, maternal anthropometry at 14–16 weeks (body mass index (BMI), height, weight, waist, hip, waist-hip ratio, waist-height ratio, arm circumference and head circumference), pulse and systolic blood pressure at 14–16 weeks. At 19–21 weeks, gestational weight gain between 14–16 and 19–21 weeks (measured in kg/week), smoking status and history of never exercising were selected. Ultrasound parameters measured at the 19–21 weeks scan included head circumference (HC), abdominal circumference (AC), femur length (FL), uterine artery Doppler resistance index (RI), and umbilical artery Doppler RI.

A group of candidate biomarkers, comprised of 7 biomarkers associated with obesity and / or with a role in glucose or lipid metabolism, were measured in samples from 14–16 weeks. Random whole blood glucose concentrations at 14–16 weeks and 19–21 weeks were also included [10]. An additional 46 biomarkers measured in samples from 14–16 weeks and previously reported in SCOPE were also explored [11]. These biomarkers were related to placentation, inflammation and angiogenesis. Of the full list of 55 biomarkers available for analysis, 10 had >40% of measurements on or below the limit of detection and therefore were excluded from further analysis. The methodology for the measurements of all biomarkers is provided in S1 Appendix and summarised in S1 Table.

### Statistical analysis

All participants with outcome data were included in the analysis. Missing data for clinical and ultrasound predictors were minimal (≤2%), except for maternal birthweight (5.2%), gestational weight gain between 14–16 and 19–21 weeks (3.0%), smoking status at 19–21 weeks (2.6%), exercise at 19–21 weeks (3.0%), average uterine artery Doppler (6.1%), and random glucose at 19–21 weeks (3.5%). Missing data were imputed for analyses using expected maximization, or for variables unrelated to other data points that had <1% missing data, single imputation was performed using the median (continuous variables) or mode (binary/categorical variables) as previously described [9]. We chose this method of imputation to allow calculation of post estimation parameters in model selection. To confirm our findings, we performed a sensitivity analysis using multiple imputation by chained equations and compared the coefficients of final prediction models between the two methods of imputation.

The dataset was randomly divided into training and validation cohorts, stratified for geographical area (Australasian centres and European centres) in a ratio of 2:1. Development of prediction models was performed using the training dataset and performance assessed in the validation dataset. Continuous factors were assessed for linearity and variation with gestational age. In total, 10 biomarkers required multiple of median (MoM) transformation (brain natriuretic peptide (BNP), fas cell surface death receptor (FAS), nephrin, plasminogen activator inhibitor 2 (PAI-2), pregnancy associated plasma protein A (PAPP-A), placental growth factor (PlGF), total cholesterol, HDL-cholesterol, LDL-cholesterol and triglycerides). All biomarkers were log transformed for analyses. Ultrasound biometry parameters (HC, AC and FL) were transformed into z-scores and uterine artery and umbilical artery Doppler RI was transformed into MoM for gestational age. Univariate analyses were performed using t-test, Mann-Whitney test or Χ^2^ test, as appropriate. Factors for model selection were chosen based on *a-priori* hypotheses except for the additional biomarkers where p<0.01 was used for inclusion.

Model selection was performed using stepwise selection based on Bayesian Information Criterion (BIC) as the stopping rule. The prediction model was developed in stages, which included different combination of groups of predictors based on applicability in clinical practice. Factors included in each model were: model 1—clinical factors at 14–16 weeks; model 2—clinical factors and candidate biomarkers at 14–16 weeks; model 3—clinical factors and ultrasound at 14–16 and 19–21 weeks; model 4—clinical factors, ultrasound and candidate biomarkers at 14–16 and 19–21 weeks; and model 5—full model including additional list of biomarkers. Performance of prediction models was assessed based on the area under the Receiver Operating Characteristic curve (AUC). The detection rate at a fixed false positive rate (FPR) of 10 and 25% was also estimated. LGA at term (birthweight above the 90^th^ centiles at or beyond 37 weeks) was the outcome used for primary analysis (univariate analysis, model development and test performance). A sensitivity analysis of model performance using birthweight at term above the 95^th^ centile as the outcome was also performed. Imputation using expected maximization was performed using “mix” package in R, version 2.9.1, (R Foundation, Vienna, Austria) and SPSS, version 24.0 (IBM Corp, Armonk, US). Statistical analysis and multiple imputation by chained equations were performed in STATA software, version 13.0 (StataCorp LP, College Station, Texas). This study has been reported in line with STROBE recommendations [13].

## Results

**Fig 1:**
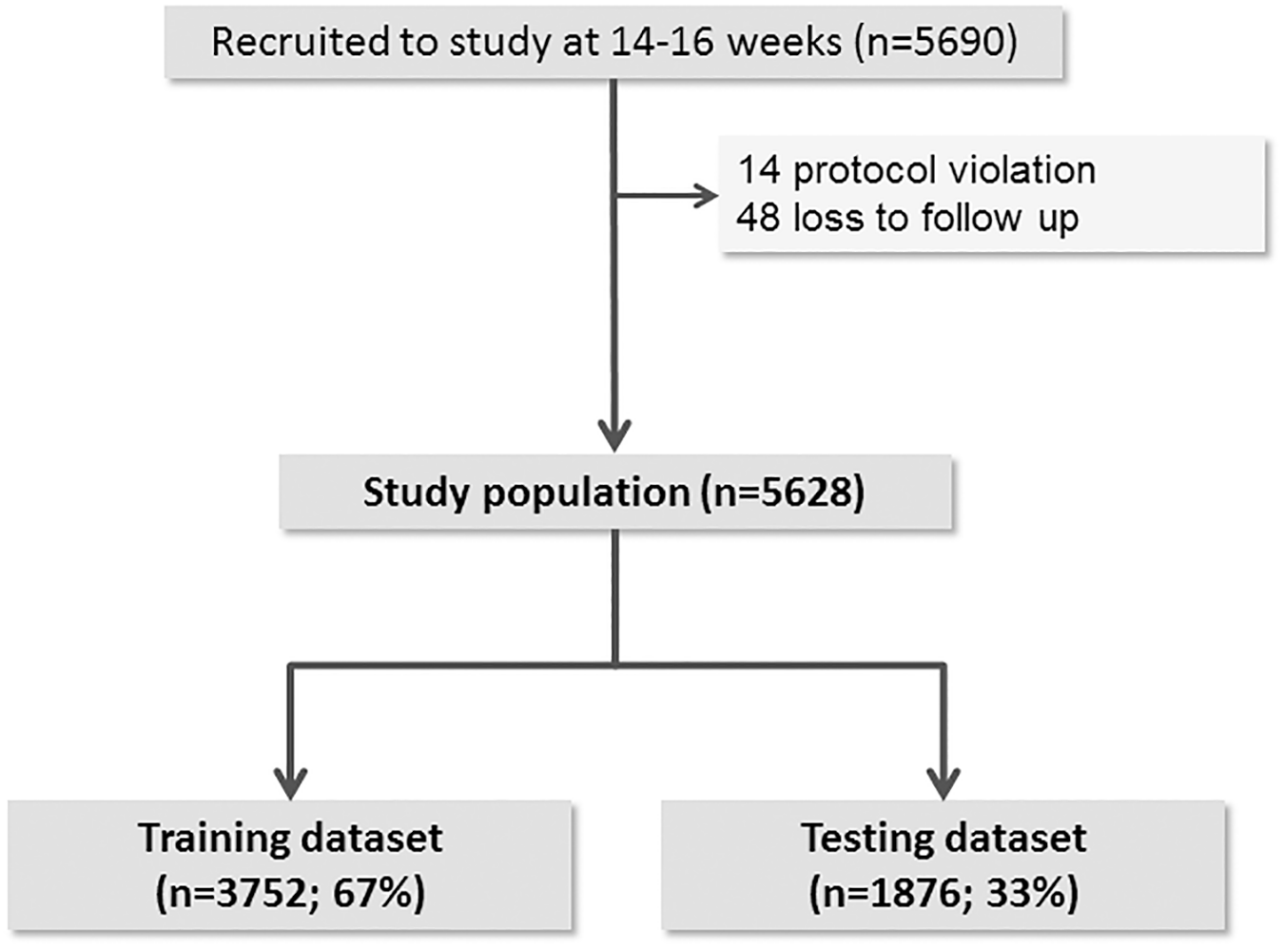
Study population.

Of the 5690 women recruited to SCOPE, 62 (1.1%) were excluded from analysis due to protocol violation or loss of follow up (Fig 1). The study population comprised 5628 women and the prevalence of LGA by customised centiles at term was 8.8% (n = 491).

The prevalence of LGA at term by customised centiles in the training (n = 3752) and validation (n = 1876) dataset was similar (8.8%, n = 331 and 8.5%, n = 160, respectively). Demographic characteristics and pregnancy outcomes of women in the training dataset are described in Table 1. Women delivering an LGA infant were more likely to develop gestational diabetes, deliver by caesarean section and have postpartum haemorrhage.

**Table 1:** Demographic characteristics and pregnancy outcomes by LGA status.

	Non-LGA at term	LGA at term	
	(N = 3421; 91.2%)	(N = 331; 8.8%)	
	Mean (SD) or n (%)	Mean (SD) or n (%)	p-value
Age (years)	28.5 (5.5)	28.9 (5.5)	0.31
Ethnicity			
European	3091 (90.4)	289 (87.3)	
Asian	100 (2.9)	10 (3.0)	0.25
Indian	80 (2.3)	14 (4.2)	
Maori / Pacific Islander	64 (1.9)	8 (2.4)	
Other	86 (2.5)	10 (3.0)	
Married/cohabiting	3092 (90.4)	306 (92.4)	0.22
Tertiary education	2840 (83.0)	279 (84.3)	0.56
Family history of DM	453 (13.2)	54 (16.3)	0.12
Gestational diabetes * †	76 (2.2)	14 (4.2)	0.02
Induction of labor †	1102 (32.8)	103 (32.2)	0.82
Mode of delivery †			
Spontaneous vaginal	1596 (46.9)	96 (29.0)	<0.001
Assisted vaginal	910 (26.7)	83 (25.1)	0.52
Elective section	287 (8.4)	51 (15.4)	<0.001
Emergency section	612 (18.0)	101 (30.5)	<0.001
Postpartum hemorrhage †	132 (4.6)	26 (9.4)	0.001
GA at delivery (wks)	39.5 (2.7)	39.8 (1.2)	0.11
Birthweight, grams †	3323 (552)	4198 (359)	<0.001
Macrosomia (>4500g) †	223 (6.5)	228 (68.9)	<0.001
Apgar<7 at 5 minutes †	49 (1.5)	1 (0.3)	0.09
NICU admission †	387 (11.3)	35 (10.6)	0.67
Severe neonatal morbidity †	102 (3.0)	13 (3.9)	0.35

Abbreviations: BP—blood pressure, DM—diabetes mellitus, GA—gestational age, LGA—large for gestational age, NICU—neonatal intensive care unit

* Women were referred for oral glucose tolerance test according to local policies. 1,300 (35%) women did not have any serum screening and this was a low risk group that had lower prevalence of cesarean section and similar prevalence of postpartum hemorrhage and NICU admission compared to women tested negative.

† Missing data for gestational diabetes (n = 14), induction of labor (n = 72), mode of delivery (n = 16), postpartum hemorrhage (n = 619), birthweight (n = 15), macrosomia (n = 15), Apgar at 5 minutes (n = 64), NICU admission (n = 14) and severe neonatal morbidity (n = 14).

Univariate comparison of pregnancy factors between LGA and non-LGA infants in the training dataset is described in S2 Table. Mothers of LGA infants had a higher birthweight, larger maternal head circumference, higher pulse and lower blood pressure at 14–16 weeks. At 19–21 weeks they were less likely to smoke and had a higher gestational weight gain between 14–16 and 19–21 weeks. Fetal HC, AC and FL z-scores at 19–21 weeks ultrasound were greater in LGA infants, and a lower uterine artery and umbilical artery RI was observed. Women who delivered LGA infants had a higher random glucose, total cholesterol and LDL-cholesterol concentration at 14–16 weeks, and higher random glucose concentration at 19–21 weeks. From the additional biomarkers, neutrophil gelatinase-associated lipocalin (NGAL), PAPP-A, and vascular endothelial growth factor receptor-1 (VEGFR1) were associated with LGA (p<0.01) and were included in the model selection process.

The prediction models developed are described in Table 2. Maternal birthweight was the only clinical factor at 14–16 weeks that was selected as a predictor in model 1. The addition of candidate biomarkers selected maternal birthweight, random glucose and LDL-cholesterol at 14–16 weeks (model 2). The model with clinical factors at 14–16 and 19–21 weeks and ultrasound included maternal birthweight, gestational weight gain between 14–16 and 19–21 weeks, fetal AC and HC z-scores on ultrasound, and uterine artery Doppler RI (model 3). The addition of candidate biomarkers measured to model 3 included random glucose at 14–16 weeks and 19–21 weeks (model 4). A complete model with clinical factors at 14–16 and 19–21 weeks, candidate and additional biomarkers and ultrasound included all the factors identified in model 4, VEGFR1 and NGAL (model 5).

**Table 2:** Description of prediction models for LGA at term in training dataset.

Predictors	Model 1 *	Model 2 *	Model 3 *	Model 4 *	Model 5 *
	OR (95%CI)	OR (95%CI)	OR (95%CI)	OR (95%CI)	OR (95%CI)
	(n = 3,752)	(n = 3,752)	(n = 3,752)	(n = 3,752)	(n = 3,752)
**Clinical factors at 14–16 weeks**					
Maternal birthweight (per 500g)	1.23 (1.11–1.37)	1.25 (1.13–1.39)	1.18 (1.06–1.31)	1.19 (1.07–1.33)	1.19 (1.06–1.32)
**Candidate biomarkers at 14–16 weeks**					
Random glucose (per 0.2 log)		1.28 (1.12–1.45)		1.23 (1.08–1.41)	1.27 (1.11–1.45)
LDL- cholesterol (per 1 log of MoM)		1.85 (1.22–2.80)			
**Clinical factors and ultrasound at 19–21 weeks**					
Gestational weight gain (per 500g/week)			1.31 (1.14–1.50)	1.32 (1.14–1.51)	1.32 (1.14–1.53)
AC Z-score at ultrasound			1.52 (1.34–1.72)	1.51 (1.34–1.71)	1.52 (1.34–1.73)
HC Z-score at ultrasound			1.38 (1.21–1.57)	1.37 (1.21–1.57)	1.40 (1.22–1.59)
Uterine artery RI (per 0.2 MoM)			0.70 (0.61–0.81)	0.69 (0.60–0.79)	0.71 (0.62–0.82)
**Candidate biomarkers at 19–21 weeks**					
Random glucose (per 0.2 log)				1.22 (1.07–1.39)	1.22 (1.07–0.39)
**Additional biomarkers at 14–16 weeks**					
VEGFR1 (log)					1.67 (1.40–2.00)
NGAL (log)					0.62 (0.48–0.81)

Abbreviation: AC—abdominal circumference, HC—head circumference, MoM—multiple of median, NGAL—neutrophil gelatinase-associated lipocalin, RI—resistance index, VEGFR1—vascular endothelial growth factor receptor type 1.

* Model 1—clinical factors at 14–16 weeks; Model 2—clinical factors and candidate biomarkers at 14–16 weeks; Model 3—clinical factors and ultrasound at 14–16 and 19–21 weeks; Model 4—clinical factors, ultrasound and candidate biomarkers at 14–16 and 19–21 weeks; Model 5—full model including additional list of biomarkers.

**Fig 2:**
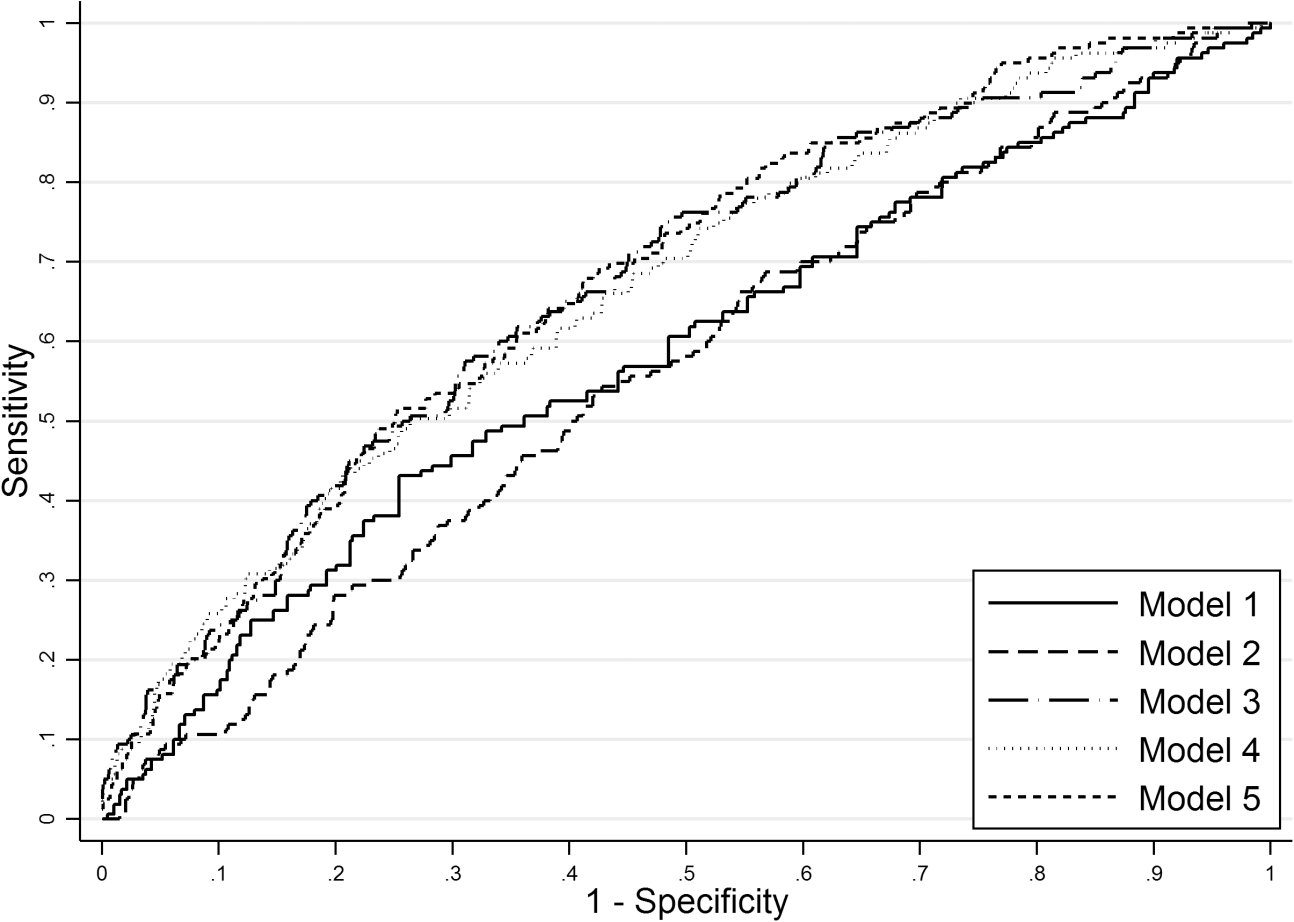
Receiver operating characteristics curve for LGA prediction models in the validation dataset. Model 1—clinical factors at 14–16 weeks; Model 2—clinical factors and candidate biomarkers at 14–16 weeks; Model 3—clinical factors and ultrasound at 14–16 and 19–21 weeks; Model 4—clinical factors, ultrasound and candidate biomarkers at 14–16 and 19–21 weeks; Model 5—full model including additional list of biomarkers.

The performance of different predictive models in training and validation datasets is described in Table 3 and the receiver operator characteristics curve in the validation dataset plotted in Fig 2. Model 1, which selected only one clinical factor at 14–16 weeks had poor performance. This was improved with the addition of clinical and ultrasound parameters at 19–21 weeks (Model 3; AUC 0.67, 0.63 to 0.71; p = 0.001 for comparison with Model 1; validation dataset). The full model including clinical factors, ultrasound and biomarkers produced an AUC of 0.69 (0.65 to 0.73; validation dataset) (Model 5), which was not statistically different from Model 3 (p = 0.21). For a fixed FPR of 10% and 25%, the detection rates (DR) in the validation dataset were 24% and 49% for model 3 and 26% and 50% for Model 5, respectively. A sensitivity analysis assessing model performance using birthweight above the 95^th^ centiles as the outcome produced very similar results (S3 Table). Similar coefficients for the five prediction models were observed in the sensitivity analysis using multiple imputation by chained equations (S4 Table).

**Table 3:** Detection rate and area under the receiver operating characteristic of the prediction models for LGA at term.

	Training dataset			Validation dataset		
Models *	10% FPR	25% FPR	AUC (95%CI)	10% FPR	25% FPR	AUC (95%CI)
1 MBW	14%	35%	0.57 (0.54–0.60)	16%	38%	0.59 (0.54–0.64)
2 MBW, gluc, and LDL (14-16w)	18%	38%	0.60 (0.57–0.63)	11%	30%	0.56 (0.52–0.61)
3 MBW, GWG, AC, HC, and UtRI (19-21w)	30%	55%	0.70 (0.67–0.73)	24%	49%	0.67 (0.63–0.71)
4 MBW, gluc (14-16w), GWG, AC, HC, UtRI, and gluc (19-21w)	33%	56%	0.72 (0.69–0.75)	26%	48%	0.66 (0.62–0.71)
5 MBW, gluc (14-16w), GWG, AC, HC UtRI, and gluc (19-21w), VEGFR1 and NGAL (14-16w)	35%	60%	0.74 (0.71–0.77)	26%	50%	0.69 (0.65–0.73)

Abbreviations: AC—fetal abdominal circumference, AUC–area under the receiver operating characteristic, gluc—glucose, GWG–gestational weight gain between 14–16 and 19–21 weeks, FPR–false positive rate, HC—fetal head circumference, LDL—LDL-cholesterol, MBW—maternal birthweight, NGAL—neutrophil gelatinase-associated lipocalin, UtRI—uterine artery resistance index, VEGFR1—vascular endothelial growth factor receptor type 1, w—weeks.

* Model 1—clinical factors at 14–16 weeks; Model 2—clinical factors and candidate biomarkers at 14–16 weeks; Model 3—clinical factors and ultrasound at 14–16 and 19–21 weeks; Model 4—clinical factors, ultrasound and candidate biomarkers at 14–16 and 19–21 weeks; Model 5—full model including additional list of biomarkers.

## Discussion

We developed a prediction model for LGA at term defined using customised birthweight centiles. Maternal birthweight, gestational weight gain between 14–16 to 19–21 weeks, fetal AC and HC z-score and uterine artery RI at the 19–21 weeks ultrasound contributed independently to the prediction of LGA. Random glucose, VEGFR1 and NGAL at 14–16 weeks, and random glucose at 19–21 weeks were also independent predictors. The performance of the full prediction model was modest with an AUC of 0.69 (0.65 to 0.73) and a detection rate of 26% and 50% for a fixed FPR of 10% and 25%, respectively.

At present, primary prevention of fetal overgrowth leading to LGA is limited by poor prediction and by the lack of effective antenatal interventions in non-GDM pregnancies [14, 15]. However, secondary prevention to avoid complications of labour and delivery has now been shown to be achievable in a well-designed randomised controlled trial [4]. In this large multi-centre trial, Boulvain *et al* reported that induction of labour at 37^+0^ to 38^+6^ weeks in pregnancies with suspected LGA infants (estimated fetal weight on ultrasound above the 95^th^ centile between 36–38 weeks) reduced the risk of shoulder dystocia and associated neonatal morbidity (RR 0.32; 95%CI 0.15–0.71) without increasing caesarean section rates (RR 0.89; 95%CI 0.72–1.09). Women were referred for ultrasound based on increased fundal height or fetal weight estimated with the Leopold manoeuvres, although the sensitivity of the screening strategy was not reported.

Studies reporting routine clinical detection of birthweight above the 90^th^ centile for gestational age have described sensitivity between 9.7% and 16.6% [5–7]. These methods include abdominal palpation with or without ultrasound. Using these methods the majority of infants who would potentially benefit from induction of labour are not identified. The clinical applicability of the prediction model reported in the present study is limited by its modest performance. Nonetheless, it has potential future value in risk stratification, as the sensitivity of 25% FPR (49%) is higher than current clinical practice. Contingency screening by mid pregnancy risk stratification, and referral of high risk women for late third trimester scan could reduce the FPR and direct resources to women at higher risk of LGA. Although one in every four women would require a third trimester scan, the addition of clinical factors in late pregnancy such as maternal weight gain could further improve the model and reduce the number of scans. Registry studies reported that late 3^rd^ trimester ultrasound has a sensitivity and specificity of 72–73% and 87–90% for LGA, respectively [16, 17]. However, this is not universal practice due to increased antenatal health care costs and utilization of ultrasound services. Further studies are required to assess effectiveness and health economic benefits of contingency screening and universal third trimester ultrasound to clarify which is the most cost-effective approach in the detection of LGA.

In contrast to previous reports predicting LGA by population centiles, in our cohort maternal anthropometric measures were not associated with LGA by customised centiles [17, 18]. This may relate to the adjustment for maternal weight and height in the estimation of customised centiles. Our prediction model was substantially driven by the ultrasound parameters at 19–21 weeks, suggesting that fetal overgrowth may be established as early as 19–21 weeks in some women. Amongst ultrasound parameters, AC z-score had the stronger association with LGA and this agrees with previous reports in which AC and estimated fetal weight at the last available scan were the best predictors of term and preterm LGA [19, 20]. Furthermore in contrast to our cohort of nulliparous women, these previous models were developed from unselected populations which included multiparous women. A previous LGA infant is a recognised risk factor for a subsequent LGA infant. However, mode of delivery in previous pregnancy will provide reassurance for management of subsequent pregnancy which limits clinical relevance of prediction in multiparous compared with nulliparous women. Lack of a past obstetric history in nulliparous women also increases the potential value of a predictive tool. The contribution of maternal anthropometrics and previous LGA are likely related to the higher AUC (0.79; 95% CI 0.79–0.79) at 19–24 weeks observed by Frick et al [17]. They have also shown that prediction is improved with ultrasound in later gestations. At 30–34 weeks, their prediction model using maternal characteristics and fetal biometry achieved an AUC of 0.85 (0.85–0.86), however only one third of their population had ultrasound at that gestation. It is likely that performance would be considerably lower if the two thirds of women without available ultrasound were accounted for. Clinical translation of their finding is limited as universal third trimester screen is not available at present in the UK and the majority of countries worldwide.

Mechanistically, elevated maternal glucose concentrations provide the traditional explanation for accelerated fetal growth which has been emphasised in a recent study using mendelian randomization, suggesting maternal BMI and blood glucose are likely to be causally associated with higher offspring birthweight [21]. In the absence of overt hyperglycaemia, maternal insulin and triglycerides may signal increased placental transport of fatty acids leading ultimately to macrosomia [22–24]. Although an independent association between glucose and LDL-cholesterol with LGA was shown in our study, the contribution to the predictive performance was minimal (Table 3). The lack of association with triglycerides may reflect the time of measurement at 14–16 weeks, which may have little relevance to later fetal growth, [25] or, alternatively, previously observed association could be explained by unmeasured confounders, as this association was also not apparent using mendelian randomisation [21]. VEGFR1 is the receptor for vascular endothelial growth factor (VEGF) and provided a mild increase in the AUC. The use of biomarkers did not improve overall performance of the prediction of LGA in early pregnancy.

SCOPE was not developed with the primary aim of early prediction of LGA but this rich dataset provides an opportunity for testing further hypotheses using this well characterised cohort with highly complete data. This cohort which is enriched with early pregnancy factors provides the opportunity to explore their contribution to the prediction of LGA. Another strength of this study is internal validation in a separate dataset of SCOPE participants, which differs from previous studies [17, 18]. A limitation is the wide variation in the screening for gestational diabetes mellitus (GDM), which was performed according to local policy in each centre. However, the prevalence of LGA associated with known GDM was small (5%) and our results were consistent in a sensitivity analysis excluding all cases of GDM. Other limitations include the gestation of biomarker measurement (14–16 weeks), which is not the time of a routine antenatal visit in many countries.

## Conclusion

In this study, we have developed a prediction model for LGA by customised centiles at term. Overall, the performance of prediction models for LGA up to 22 weeks is limited and the addition of biomarkers does not improve performance. Other strategies such as contingency screening, with risk stratification at 20 weeks and tailored ultrasound assessment in the late third trimester, or universal third trimester ultrasound screening are likely to improve antenatal detection of LGA infants. Further studies need to explore benefits and health economic costs of these different screening strategies.

## Supporting information

S1 Appendix Methodology for measurement of biomarkers. (DOC)

S1 Table List of biomarkers measured at 14–16 weeks gestation and the assay method. (DOC)

S2 Table Description of factors explored for association with term LGA at 14–16 and 19–21 weeks in the training dataset. (DOC)

S3 Table Detection rate and area under the receiver operating characteristic curve of the prediction models for birthweight above the 95th centile. (DOC)

S4 Table Sensitivity analysis with the description of prediction models for LGA at term in training dataset using multiple imputation with chained equation. (DOCX)
