# Supplementary material for: Clinical, ultrasound and molecular biomarkers for early prediction of large for gestational age infants in nulliparous women: An international prospective cohort study
Source: PLoS One. 2017 Jun 1;12(6):e0178484. doi: 10.1371/journal.pone.0178484 (PMC5453528; doi:10.1371/journal.pone.0178484)
Supplement: S1 Appendix — (DOC) [file pone.0178484.s001.doc]

**Clinical Risk Factors, Ultrasound and Biomarkers for Early Prediction of Large for Gestational Age Infants in Nulliparous Women: an International Prospective Cohort Study.**

**S1 Appendix. Methodology for measurement of biomarkers**

From the 9 candidate biomarkers, 3 (total cholesterol, HDL-cholesterol and triglycerides) were measured using Beckman Unicel Syncron DxC (Beckman Coulter, Inc., Brea, CA 92821 USA). Measurements were not repeated for participants with previously available results for these analytes, which were performed using COBAS (Roche Diagnostics Ltd., CH-6343 Rotkreuz, Switzerland).^1^ Both methods used the same reactions. The LDL-cholesterol was calculated using the Friedewald Formula. In addition, random whole blood glucose at 14-16 weeks and at 19-21 weeks was obtained from finger prick blood using Optimum Glucometer or MediSense Optium Xceed Glucometer (Abbott Diabetes Care Ltd, Witney, Oxon, OX29 0YL, UK).

The remaining three additional biomarkers (adiponectin, insulin and placental growth hormone) were measured using the same platform used for measurement of the 46 previously reported biomarkers^2^ with the methodology below.

Mouse derived recombinant Fab(s), generated by unique phage display technology (Alere, San Diego), were used in several immunoassay formats for the measurement of biomarker concentrations. These reagents were optimized for use in Luminex xMap technology (Alere, San Diego) or micro-titer plate ELISAs. Once optimized for format, a small subset of clinical patient samples were used to determine the final reagents (pairings or single recombinant Fabs) to use in the full cohort screening.

Luminex assays utilize magnetic beads that have a unique ratio of two dyes that are used to distinguish the identity of each bead, thereby enabling the multiplexing of assays within the same well. The Luminex sandwich assay format uses a recombinant Fab conjugated to a magnetic bead as the capture and a biotin-conjugated recombinant Fab as the assay detection. Recombinant Fab conjugated magnetic beads are added to the plate and washed. Sample is then incubated with the beads, followed by incubation with the detection antibody. The plate is washed, incubated with strepavidin labeled phycoerythrin, washed and then read using a Luminex 200 reader. The Luminex competitive assay format uses a recombinant Fab conjugated to the bead and a biotin-conjugated antigen as the assay detection. Recombinant Fab conjugated magnetic beads are added to the plate and washed. The sample and detection reagent are premixed, added simultaneously and then incubated. The final steps are as described for the Luminex sandwich assay.

The micro-titer ELISA assays use a streptavidin coated plate and biotin or fluorescein conjugated recombinant Fabs. The ELISA sandwich assay uses a biotin-conjugated recombinant Fab as the capture and a fluorescein-conjugated recombinant Fab as the detection antibody. Capture antibody is coated on the plate, incubated, washed and sample added. After sample incubation, the plate is washed and then incubated with detection antibody. Following washing, the plate is incubated with anti-fluorescein antibody conjugated to alkaline phosphatase, washed, fluorescent substrate added and then read using a Tecan infinite F200 reader.

The ELISA competitive assay uses a biotin-conjugated antigen as the capture and a fluorescein-conjugated recombinant Fab as the detection antibody. The plate is coated with capture antibody, incubated and washed. Addition of sample is immediately followed by addition of the detection antibody and incubated. The final steps are the same as the ELISA sandwich.

Each assay uses an 8 point dose curve prepared gravimetrically in EDTA plasma or buffer.

**References**

1. Fyfe EM, Rivers KS, Thompson JM, Thiyagarajan KP, Groom KM, Dekker GA, et al. Elevated maternal lipids in early pregnancy are not associated with risk of intrapartum caesarean in overweight and obese nulliparous women. BMC Pregnancy Childbirth. 2013;13:143

2. Kenny LC, Black MA, Poston L, Taylor R, Myers JE, Baker PN, et al. Early pregnancy prediction of preeclampsia in nulliparous women, combining clinical risk and biomarkers: the Screening for Pregnancy Endpoints (SCOPE) international cohort study. Hypertension. 2014;64(3):644-52.
