# Supplementary material for: Clinical, ultrasound and molecular biomarkers for early prediction of large for gestational age infants in nulliparous women: An international prospective cohort study
Source: PLoS One. 2017 Jun 1;12(6):e0178484. doi: 10.1371/journal.pone.0178484 (PMC5453528; doi:10.1371/journal.pone.0178484)
Supplement: S1 Table — (DOC) [file pone.0178484.s002.doc]

**S1 Table**. List of biomarkers and the assay method.

| **Biomarker** | **Assay method** | **Included in analysis** | **Convert to MoM** |
| --- | --- | --- | --- |
| **Candidate biomarkers at 14-16 weeks** | |  |  |
| Adiponectin | Luminex Competitive | Yes |  |
| Cholesterol (total) | Enzymatic Colorimetric | Yes | Yes |
| Glucose (random) | Electrochemical sensor | Yes |  |
| HDL-cholesterol | Enzymatic Colorimetric | Yes |  |
| Insulin | Luminex Sandwich | Yes |  |
| LDL-cholesterol | Calculated (Friedewald Formula) | Yes | Yes |
| Placental growth hormone (PlGH) | Luminex Sandwich | Exclude LOD * | Yes |
| Triglycerides | Enzymatic Colorimetric | Yes | Yes |
| **Candidate biomarker at 19-21 weeks** | |  |  |
| Glucose (random) | Electrochemical sensor | Yes |  |
| **Additional biomarkers at 14-16 weeks** | |  |  |
| Adam-9 (Disintegrin and metalloproteinase domain-containing protein 9) | Luminex Sandwich | Exclude LOD * |  |
| Angiogenin | Luminex Competitive | Yes |  |
| Arginase-1 | Luminex Sandwich | Yes |  |
| Arginase-2 | Luminex Sandwich | Yes |  |
| Atrial natriuretic peptide (ANP)-propeptide | Luminex Sandwich | Yes |  |
| Big Endothelin-1 | Luminex Sandwich | Exclude LOD * |  |
| Brain natriuretic peptide (BNP) | Luminex Sandwich | Yes | Yes |
| C-Met | Luminex Sandwich | Yes |  |
| C-reactive protein (CRP) | Luminex Competitive | Yes |  |
| C-X-C motif chemokine 10 (CXCL 10) | Luminex Sandwich | Yes |  |
| Carboxypeptidase A4 (CPA-4) precursor | Luminex Sandwich | Yes |  |
| Caspase-3 | Luminex Sandwich | Yes |  |
| Chemokine (C-C motif) ligand 23 (CCL23) | Luminex Sandwich | Yes |  |
| Cystatin C | ELISA Competitive | Yes |  |
| Elafin | Luminex Competitive | Yes |  |
| Endoglin | ELISA Sandwich | Yes |  |
| Endothelial cell-selective adhesion molecule (ESAM-1) | Luminex Sandwich | Yes |  |
| Ephrin-receptor-2 | Luminex Sandwich | Exclude LOD * |  |
| Factor inhibiting hypoxia inducible factor 1α (FIH) | Luminex Sandwich | Yes |  |
| Fas cell surface death receptor (FAS) | Luminex Sandwich | Yes | Yes |
| Fas ligand (Fas L)-soluble | Luminex Sandwich | Exclude LOD * |  |
| Intercellular adhesion molecule-1 (ICAM-1) | Luminex Competitive | Yes |  |
| Interleukin 1 receptor antagonist (IL-1ra) | Luminex Sandwich | Yes |  |
| Kunitz type protease inhibitor 2 (HAI-2) | Luminex Sandwich | Yes |  |
| Leptin | Luminex Sandwich | Yes |  |
| Leptin receptor | Luminex Sandwich | Yes |  |
| Macrophage migration inhibitory factor (MIF) | Luminex Sandwich | Yes |  |
| Matrix metalloproteinase-9 (MMP-9) | Luminex Sandwich | Yes |  |
| Nephrin | Luminex Sandwich | Yes | Yes |
| Neutrophil gelatinase-associated lipocalin (NGAL) | ELISA Sandwich | Yes |  |
| Pentraxin-3 | Luminex Sandwich | Exclude LOD * |  |
| Periostin | Luminex Sandwich | Yes |  |
| Placental growth factor (PlGF) | Luminex Sandwich | Yes | Yes |
| Plasminogen activator inhibitor 1 (PAI-1) | Luminex Sandwich | Exclude LOD * |  |
| Plasminogen activator inhibitor 2 (PAI-2) | Luminex Sandwich | Yes | Yes |
| Podocalyxin | Luminex Sandwich | Exclude LOD * |  |
| Pregnancy associated plasma protein A (PAPP-A) | Luminex Sandwich | Yes | Yes |
| Procalcitonin (PCT) | Luminex Sandwich | Exclude LOD * |  |
| ST2 | Luminex Sandwich | Yes |  |
| TIMP metallopeptidase inhibitor 1 (TIMP-1) | Luminex Competitive | Yes |  |
| Transforming growth factor (TGF) β receptor 2 | Luminex Sandwich | Yes |  |
| Tumour necrosis factor receptor 1a (TNFR1a) | Luminex Sandwich | Yes |  |
| Vascular endothelial growth factor C (VEGF-C) | Luminex Sandwich | Yes |  |
| Vascular endothelial growth factor receptor-1 (VEGFR1) | Luminex Sandwich | Yes |  |
| Visfatin | Luminex Sandwich | Exclude LOD * |  |
| WAP four disulfide core domain protein 2 (HE4) | Luminex Sandwich | Yes |  |

* Excluded from analysis as the majority of measurements were below the limit of detection (LOD) of the assay.
