# Supplementary material for: Clinical, ultrasound and molecular biomarkers for early prediction of large for gestational age infants in nulliparous women: An international prospective cohort study
Source: PLoS One. 2017 Jun 1;12(6):e0178484. doi: 10.1371/journal.pone.0178484 (PMC5453528; doi:10.1371/journal.pone.0178484)
Supplement: S2 Table — (DOC) [file pone.0178484.s003.doc]

**S2 Table.** Description of factors explored for association with term LGA at 14-16 and 19-21 weeks in the training dataset.

|  | **Non-LGA at term** | **LGA at term** |  |
| --- | --- | --- | --- |
|  | **(N=3421; 91.2%)** | **(N=331; 8.8%)** |  |
|  | **Mean (SD) or n (%)** | **Mean (SD) or n (%)** | **p** |
| **Clinical factors at 14-16 weeks** | |  |  |
| Maternal birthweight | 3296 (553) | 3422 (601) | <0.001 |
| Maternal preterm birth | 154 (4.5) | 14 (4.2) | 0.84 |
| FH of diabetes | 453 (13.2) | 54 (16.3) | 0.12 |
| BMI | 25.3 (4.9) | 25.5 (4.9) | 0.39 |
| Height (cm) | 165.0 (6.2) | 164.5 (6.8) | 0.20 |
| Weight (kg) | 68.9 (14.1) | 69.2 (14.5) | 0.64 |
| Waist (cm) | 84.0 (11.1) | 84.8 (12.1) | 0.21 |
| Hip (cm) | 101 (11) | 102 (11) | 0.34 |
| Waist hip ratio | 0.8 (0.1) | 0.8 (0.1) | 0.46 |
| Waist height ratio | 0.5 (0.1) | 0.5 (0.1) | 0.11 |
| Arm circumference (cm) | 28.1 (3.9) | 28.2 (3.9) | 0.68 |
| Head circumference (cm) | 55.7 (1.7) | 56.0 (1.7) | 0.004 |
| Pulse | 77 (11) | 79 (11) | 0.03 |
| Systolic blood pressure (mmHg) | 107 (11) | 106 (11) | 0.02 |
| Never exercised | 302 (8.8) | 36 (10.9) | 0.20 |
| **Clinical factors at 19-21 weeks** | |  |  |
| Gestational weight gain (kg/week) | 0.5 (0.4) | 0.6 (0.4) | <0.001 |
| Smoking | 361 (10.6) | 22 (6.6) | 0.03 |
| Never exercised | 267 (7.8) | 22 (6.6) | 0.45 |
| **Ultrasound at 19-21 weeks** | |  |  |
| HC z-score | -0.04 (0.98) | 0.46 (1.03) | <0.001 |
| AC z-score | -0.05 (0.96) | 0.55 (1.17) | <0.001 |
| FL z-score | -0.02 (0.99) | 0.24 (1.06) | <0.001 |
| Mean uterine artery RI * | 1.01 (0.18) | 0.95 (0.16) | <0.001 |
| Umbilical artery RI * | 1.00 (0.09) | 0.99 (0.08) | 0.03 |
| **Candidate biomarkers** | |  |  |
| *14-16 weeks* |  |  |  |
| Random glucose (mmol/L) | 5.3 (1.0) | 5.5 (1.0) | <0.001 |
| Total cholesterol * † | 1.00 (0.90 - 1.10) | 1.02 (0.92 - 1.13) | 0.006 |
| HDL cholesterol * † | 1.00 (0.87 - 1.15) | 1.01 (0.84 - 1.15) | 0.72 |
| LDL cholesterol * † | 0.99 (0.83 - 1.18) | 1.04 (0.85 - 1.23) | 0.008 |
| Triglycerides † ‡ | 0.99 (0.79 - 1.26) | 1.02 (0.81 - 1.29) | 0.07 |
| Adiponectin (ng/ml) † | 4345 (3260 - 5598) | 4290 (3207 - 5415) | 0.43 |
| Insulin (µIU/ml) † | 16.4 (9.5 - 27.4) | 16.4 (9.7 - 28.9) | 0.89 |
| *19-21 weeks* |  |  |  |
| Random glucose (mmol/L) | 5.4 (1.0) | 5.6 (1.1) | <0.001 |
| **Additional biomarkers** | |  |  |
| *14-16 weeks* | |  |  |
| NGAL (ng/ml) † | 40.3 (30.8 - 53.5) | 38.5 (28.7 - 48.9) | 0.002 |
| PAPP-A * † | 0.97 (0.58 - 1.73) | 1.21 (0.64 – 1.99) | <0.001 |
| VEGFR1 (ng/ml) † | 0.32 (0.21 - 0.52) | 0.40 (0.26 - 0.63) | <0.001 |

Abbreviations: AC - abdominal circumference, BMI - body mass index, FH - family history, FL - femur length, HC - head circumference, LGA - large for gestational age, NGAL - neutrophil gelatinase-associated lipocalin, PAPP-A - pregnancy associated plasma protein A, RI - resistance index, VEGFR1 - vascular endothelial growth factor receptor type 1.

* Multiple of Median (MoM) for gestational age.

† Median (IQR) shown and Mann Whitney test used.
