# Supplementary material for: Clinical, ultrasound and molecular biomarkers for early prediction of large for gestational age infants in nulliparous women: An international prospective cohort study
Source: PLoS One. 2017 Jun 1;12(6):e0178484. doi: 10.1371/journal.pone.0178484 (PMC5453528; doi:10.1371/journal.pone.0178484)
Supplement: S3 Table — (DOC) [file pone.0178484.s004.doc]

**S3 Table. Detection rate and area under the receiver operating characteristic curve of the prediction models for birthweight above the 95^th^** centile.

|  | **Training dataset** | | | **Validation dataset** | | |
| --- | --- | --- | --- | --- | --- | --- |
| **Models** * | **10% FPR** | **25% FPR** | **AUC (95%CI)** | **10% FPR** | **25% FPR** | **AUC (95%CI)** |
| 1 MBW | 15% | 35% | 0.57 (0.53 - 0.62) | 17% | 38% | 0.58 (0.51 - 0.64) |
| 2 MBW, gluc, and LDL (14-16w) | 19% | 41% | 0.61 (0.57 - 0.65) | 10% | 29% | 0.53 (0.46 - 0.59) |
| 3 MBW, GWG, AC, HC, and UtRI (19-21w) | 34% | 59% | 0.74 (0.70 - 0.77) | 29% | 51% | 0.69 (0.63 - 0.75) |
| 4 MBW, gluc (14-16w), GWG, AC, HC, UtRI, and gluc (19-21w) | 37% | 63% | 0.75 (0.72 - 0.79) | 26% | 50% | 0.67 (0.61 - 0.73) |
| 5 MBW, gluc (14-16w), GWG, AC, HC, UtRI, and gluc (19-21w), VEGFR1 and NGAL (14-16w) | 40% | 66% | 0.78 (0.74 - 0.81) | 27% | 54% | 0.70 (0.64 - 0.75) |

Abbreviations: AC - fetal abdominal circumference, AUC – area under the receiver operating characteristic, gluc - glucose, GWG – gestational weight gain between 14-16 and 19-21 weeks, FPR – false positive rate, HC - fetal head circumference, LDL - LDL-cholesterol, MBW - maternal birthweight, NGAL - neutrophil gelatinase-associated lipocalin, UtRI - uterine artery resistance index, VEGFR1 - vascular endothelial growth factor receptor type 1, w - weeks’.

* Model 1 - clinical factors at 14-16 weeks; Model 2 - clinical factors and candidate biomarkers at 14-16 weeks; Model 3 - clinical factors and ultrasound at 14-16 and 19-21 weeks; Model 4 - clinical factors, ultrasound and candidate biomarkers at 14-16 and 19-21 weeks; Model 5 - full model including additional list of biomarkers.
