# Supplementary material for: Clinical, ultrasound and molecular biomarkers for early prediction of large for gestational age infants in nulliparous women: An international prospective cohort study
Source: PLoS One. 2017 Jun 1;12(6):e0178484. doi: 10.1371/journal.pone.0178484 (PMC5453528; doi:10.1371/journal.pone.0178484)
Supplement: S4 Table — (DOCX) [file pone.0178484.s005.docx]

**Table S4. Sensitivity analysis of prediction models using multiple imputation.**

| **Predictors** | **Model 1** * | **Model 2** * | **Model 3** * | **Model 4** * | **Model 5** * |
| --- | --- | --- | --- | --- | --- |
|  | **OR (95%CI)** | **OR (95%CI)** | **OR (95%CI)** | **OR (95%CI)** | **OR (95%CI)** |
|  | **(n=3,752)** | **(n=3,752)** | **(n=3,752)** | **(n=3,752)** | **(n=3,752)** |
| **Clinical factors at 14-16 weeks** |  |  |  |  |  |
| Maternal birthweight (per 500g) | 1.26 (1.14 - 1.41) | 1.28 (1.15 - 1.43) | 1.20 (1.08 - 1.34) | 1.22 (1.09 - 1.36) | 1.21 (1.08 - 1.35) |
| **Candidate biomarkers at 14-16 weeks** | |  |  |  |  |
| Random glucose (per 0.2 log) |  | 1.28 (1.12 - 1.45) |  | 1.23 (1.08 - 1.41) | 1.28 (1.11 - 1.45) |
| LDL- cholesterol (per 1 log of MoM) |  | 1.85 (1.23 - 2.80) |  |  |  |
| **Clinical factors and ultrasound at 19-21 weeks** | |  |  |  |  |
| Gestational weight gain (per 500g/week) |  |  | 1.31 (1.13 - 1.50) | 1.32 (1.14 - 1.52) | 1.32 (1.14 - 1.52) |
| AC Z-score at ultrasound |  |  | 1.52 (1.35 - 1.72) | 1.52 (1.34 - 1.72) | 1.53 (1.35 - 1.73) |
| HC Z-score at ultrasound |  |  | 1.37 (1.21 - 1.56) | 1.37 (1.20 - 1.56) | 1.39 (1.22 - 1.59) |
| Uterine artery RI (per 0.2 MoM) |  |  | 0.70 (0.61 - 0.81) | 0.69 (0.60 - 0.80) | 0.71 (0.62 - 0.82) |
| **Candidate biomarkers at 19-21 weeks** | |  |  |  |  |
| Random glucose (per 0.2 log) | |  |  | 1.20 (1.06 - 1.36) | 1.20 (1.05 - 1.36) |
| **Additional biomarkers at 14-16 weeks** | |  |  |  |  |
| VEGFR1 (log) |  |  |  |  | 1.66 (1.39 - 1.99) |
| NGAL (log) |  |  |  |  | 0.63 (0.48 - 0.81) |

Abbreviation: AC - abdominal circumference, HC - head circumference, MoM - multiple of median, NGAL - neutrophil gelatinase-associated lipocalin, RI - resistance index, VEGFR1 - vascular endothelial growth factor receptor type 1.

* Model 1 - clinical factors at 14-16 weeks; Model 2 - clinical factors and candidate biomarkers at 14-16 weeks; Model 3 - clinical factors and ultrasound at 14-16 and 19-21 weeks; Model 4 - clinical factors, ultrasound and candidate biomarkers at 14-16 and 19-21 weeks; Model 5 - full model including additional list of biomarkers.
